# Dung-Induced Soil Microbial Community Coalescence Driven by Different Dung Sources: Impacts on Community Shifts and Assembly Mechanisms in Grassland Soils

**DOI:** 10.3390/microorganisms14071493

**Published:** 2026-07-08

**Authors:** Jie Yang, Qi Zhang, Bobo Wang, Fabrice Ndayisenga, Zhisheng Yu

**Affiliations:** 1 School of Water Resources and Environment, China University of Geosciences (Beijing), Beijing 100083, China; 2 College of Resources and Environment, University of Chinese Academy of Sciences, Beijing 100049, China; 3 RCEES-IMCAS-UCAS Joint-Lab of Microbial Technology for Environmental Science, Beijing 100085, China

**Keywords:** microbial structure, co-occurrence network, community assembly, dung deposition, grassland management

## Abstract

The overall influences of grazing practice on soil microbial community shifts have received considerable attention, but how dung-induced community coalescence affects soil microbial diversity, structure, interaction and the underlying assembly mechanism remains unclear. To address this, we investigated soil microbial community alterations in response to dung deposition, which included three different dung sources in a single grassland. The results indicated that dung deposition by different livestock species had varying impacts on soil microbial community diversity and structure, with cattle dung associated with the largest observed shifts in soil microbial diversity and community structure in this study. The structure of the soil microbial community was strongly associated with multiple edaphic properties (e.g., pH and nutrient content), but these correlations were reshaped by dung deposition in a dung-source-dependent manner. In addition, dung deposition consistently reduced the complexity and robustness of the co-occurrence network across different dung sources, and the strongest alterations in the network were found in shallow soils (0–20 cm). Null model analysis suggests that dung deposition improved the proportional contribution of the stochastic process in bacterial and fungal communities and conversely increased the deterministic process in the archaeal community, implying distinct assembly mechanisms of those microbial domains to the disturbance induced by dung deposition. These results highlight the source effect of dung deposition on the diversity and structure of soil microbial communities, as well as the domain-dependence of dung deposition on the assembly mechanism. The findings suggest that multispecies grazing is associated with distinct dung-induced microbial community shifts, highlighting the need for future research that explicitly incorporates dung source as a variable in grassland soil microbial assessments.

## 1. Introduction

Grassland ecosystems cover an area of 52.5 million km^2^ globally, providing multiple ecosystem services, such as carbon storage, climate mitigation and pollination [1,2,3]. However, anthropogenic activities and associated habitat destruction are increasingly impairing ecosystem functions and altering resource availability and cycling [4]. The impact of biodiversity change on ecosystem functioning has spurred extensive research examining the effects of land-use intensification on grassland ecosystem biodiversity and community structure [5,6,7]. This concern is particularly acute for soil biodiversity, which remains largely invisible and understudied despite its essential roles in ecosystem functioning. As anthropogenic pressures intensify, soil microbial diversity may be lost before adequate characterization—a phenomenon emblematic of the ongoing biodiversity crisis [8]. As the most extensive land-use practice, grazing and associated deposition of livestock dung are considered major drivers of biotic and abiotic changes in native grasslands, often influencing soil properties and diversity patterns. For instance, a global synthesis of grazing experiments from natural grasslands suggested that the effects of grazing on biodiversity depended on climate types (dry and wet areas) and grazing patterns (i.e., grazing intensity, livestock type, and grazing season) [9]. Understanding the impacts of grazing on grassland biodiversity and structural shifts is critical for predicting community development trajectories and understanding associated shifts in ecosystem functions (e.g., grassland productivity and carbon sequestration) in response to multiple grazing patterns.

Dung deposition represents a key mechanism by which grazing influences grassland biodiversity, and it can mediate community structure through directly altering ecosystem nutrient inputs and local soil properties [10]. Although the influences of grazing practices on biotic communities have been well-documented [11,12,13,14], particularly regarding aboveground vegetation (e.g., species diversity and productivity) [15,16,17], how these communities respond to dung deposition—as a process separate from grazing per se—remains largely unexplored. Dung deposition not only adds nutrients but also introduces alien microorganisms, potentially triggering a community coalescence between dung-derived and soil-resident microbes [18]. Such community coalescence induced by dung deposition will regulate population dynamics (including recruitment, growth and mortality) and species interactions, ultimately mediating profound changes in both below- and aboveground community structure as well as grassland ecosystem functioning [19,20,21].

The outcomes of dung-induced community coalescence may theoretically depend on dung source, coalescence frequency, biotic interactions, and soil abiotic factors [21,22,23,24]. However, most previous studies have used a single dung source or compared dung from different management histories/sites [21,25]. Consequently, the differential effects associated with various dung sources (including microbial taxa as well as physicochemical properties) on soil microbial community structure and their underlying mechanisms remain largely unexplored. The changes in microbial diversity, community structure, and interspecies interactions induced by community coalescence determine their functional capacity (e.g., greenhouse gas emissions, nutrient cycling, plant protection) [21,26,27]. Nevertheless, unravelling the microbial assembly mechanism during dung-induced coalescence is essential for predicting soil community responses. Two primary processes—deterministic and stochastic—jointly govern community assembly, but their contributions vary with environmental drivers [28,29,30]. Although knowledge of soil microbial assembly under various anthropogenic activities is growing [31,32], how dung deposition influences the balance between deterministic and stochastic processes, and how these processes structure microbial communities, remains unknown. Furthermore, dung deposition occurs on the soil surface, yet most studies have focused on shallow soil layers. Whether the effects of dung-induced coalescence penetrate deeper soil horizons and whether microbial responses differ among soil depths have rarely been examined.

To address these gaps, we conducted a comparative study in the Xilingol steppe, a geographical region in central-east Inner Mongolia, China. This site features multiple livestock types (Ujimqin sheep, Luxi cattle, and Arabian horses) that graze together under consistent grazing practices, local geology, and climate. Adjacent soils without dung deposition served as controls. We evaluated soil microbial communities (bacteria, fungi, archaea) from seven depths (0–100 mm). We hypothesized that: (i) dung deposition-induced community coalescence will alter soil microbial community structure, but the responses will differ among dung sources and soil depths, and (ii) dung deposition will decrease the complexity of co-occurrence patterns, and this effect will vary substantially among dung sources. We also quantified the assembly mechanisms (deterministic vs. stochastic) for each microbial domain. The findings are expected to enhance mechanistic understanding of dung-driven community coalescence and to provide a microbial ecological perspective that may serve as a reference for future hypothesis-driven management studies.

## 2. Materials and Methods

### 2.1. Study Area, Sample Collection and Edaphic Analysis

This study was conducted in the Xilingol steppe grassland (44°01′ N, 116°24′ E) of central-east Inner Mongolia, China. The region has a mean annual temperature of 5.1 °C and precipitation of 195 mm. The study area is characterized by flat and open terrain at an elevation of approximately 1100 m. The zonal soil of the region—i.e., soils whose development is determined primarily by climate and vegetation—is classified as calcareous chestnut soil (Calcic Kastanozem), a sandy loam developed on Quaternary loess parent material. This soil type is further divided into two subtypes: typical chestnut soil and dark chestnut soil. In contrast, intrazonal saline–alkali soils occur in some low-lying areas, where local topographic conditions override the zonal climatic signal. Given the deep groundwater table (>30 m), soil moisture is dependent on precipitation. The dominant plant species herein are *Leymus chinensis*, *Agropyron cristatum*, and *Stipa grandis*. The study site has been under long-term grazing by three livestock species (Ujimqin sheep, Luxi cattle, and Arabian horses) with a consistent grazing practice, ensuring comparable background conditions for investigating dung deposition effects. The soil samples covered by livestock dung (with dung retention time of ~40 days) and adjacent uncovered soils were collected from seven different depths (three replicate plots for each depth) with a steel probe, composed of surface soil samples (0–5 cm below ground), shallow-soil samples (5–10 cm and 10–20 cm), intermediate-depth soil samples (20–30 cm, 30–50 cm, and 50–70 cm), and deep-soil samples (70–100 cm). Fresh dung and air-dried dung pats were also aseptically collected for each livestock species. All samples were labelled and transferred to the laboratory in a dry ice box.

The edaphic properties of the samples were measured as described by Zhao et al. (2021) [33]. Soil total carbon and total nitrogen were measured on a Vario EL III Elemental Analyzer (Elementar, Hanau, Germany), while soil total phosphorus was measured by a US-VIS spectrophotometer (AA3, SEAL analytical, Norderstedt, Germany). The concentrations of nitrate and ammonium nitrogen were determined by extraction with 2M KCl, followed by analysis on a continuous-flow ion auto-analyzer (SEAL Analytical GmbH, Norderstedt, Germany). Soil pH was measured by a pH meter (Starter 3100, Ohaus Instruments, Changzhou, China) with a 1:5 soil–water ratio (ISO 10390:2021 [34]). Soil moisture was gravimetrically measured by drying the soil samples at 105 °C to a constant weight. The soil C:N ratio was calculated from the measured contents of soil total carbon and total nitrogen.

### 2.2. DNA Extraction, PCR and High-Throughput Sequencing

Genomic DNA was extracted with a MoBio PowerSoil kit (MoBio Laboratories, Carlsbad, CA, USA) following the manufacturer’s instructions. Prior to downstream analysis, a composite DNA sample for each depth was generated by pooling the triplicate extracts in the same amounts. Primers targeting the bacterial 16S rRNA gene (338F: 5′-ACTCCTACGGGAGGCAGCAG-3′ and 806R 5′-GGACTACHVGGGTWTCTAAT-3′) and those targeting the archaeal 16S rRNA gene (524F-10-ext: 5′-TGYCAGCCGCCGCGGTAA-3′ and arch958R: 5′-YCCGGCGTTGAVTCCAATT-3′) were used to construct prokaryotic amplicon libraries [35,36]. Primers targeting the fungal ITS region (ITS1F: 5′-CTTGGTCATTTAGAGGAAGTAA-3′ and ITS2R: 5′-GCTGCGTTCTTCATCGATGC-3′) were used to create fungal libraries [37]. Each DNA sample was amplified in three replicates, including a negative control with 1 μL of sterile water to monitor potential PCR reagent contamination. The PCR amplification was conducted under the following conditions: initial denaturation at 95 °C for 3 min; execution of 35 cycles of PCR (95 °C for 30 s, 55 °C for 30 s, 72 °C for 45 s); and ending with a final extension at 72 °C for 10 min. The amplicons from three independent reactions per sample were pooled for library construction to mitigate the effects of potential early-cycle PCR errors. The final PCR products were sent for sequencing on the Illumina MiSeq PE300 platform at Majorbio Bio-pharm Technology Co., Ltd. (Shanghai, China).

### 2.3. Sequence Data Analysis

Prior to analysis, raw sequences were quality-filtered using Trimmomatic to remove low-quality reads (score < 30) and those containing ambiguous bases (“N”) [38] to minimize the impacts of random sequencing errors. The remaining sequences were further processed to discard reads with primer or barcode sequences, those shorter than 200 bp or longer than 550 bp, and identified chimeras. Subsequently, the high-quality sequences were trimmed and taxonomically classified against the Silva database using the RDP classifier [39]. Finally, operational taxonomic units (OTUs) were clustered at a 97% similarity threshold with USEARCH [40].

### 2.4. Bioinformatic and Statistical Analysis

We normalized all samples to an equal sequencing depth via random subsampling to enable unbiased comparisons of microbial diversity. Subsequently, the bacterial, fungal, and archaeal libraries contained 17,247, 40,388, and 33,105 high-quality sequences per sample, respectively. The alpha diversity of soil bacterial, fungal and archaeal communities was assessed. For each community, we calculated the observed number of OTUs, Chao 1 richness estimator, phylogenetic diversity, and Shannon, Simpson and ACE indexes using Mothur [41]. The microbial community composition in different dung-covered soils and uncovered soils, together with corresponding livestock dung, was visualized using an average relative abundance. The dissimilarity in soil microbial community structure among the dung-covered and uncovered soils was analyzed by principal coordinates analysis (PCoA), and the significance of community structure among them was assessed using permutational multivariate analysis of variance (PERMANOVA) and multiple response permutation procedure (MRPP).

A network analysis was conducted to reveal the impacts of dung deposition on the soil microbial co-occurrence patterns. Only representative OTUs (>ten sequences within each dung-covered or uncovered soil) were used to conduct the following analysis. We defined a robust co-occurrence relationship as one with a Spearman’s correlation coefficient *ρ* > 0.6 and a statistically significant *p* < 0.001. We calculated eight network indexes to describe the topological features of these networks, including average path length, average clustering coefficient, average degree, network density, positive co-occurrence percentage, modularity, and total nodes and links. We quantified network robustness as the decline rate of natural connectivity during simulated node removal [42]. Network visualization was conducted using the Gephi (v0.11.2) platform [43]. Correlations between microbial community profiles and environmental factors were determined by Pearson’s correlation test and Mantel analysis, and the results were visualized using ggcor (v0.9.8.1) in R (v4.0.3).

The assembly mechanisms of the soil microbial community were assessed using a null model approach following Stegen et al. (2013) [44]. Pairwise comparisons were grouped by dung source. Specifically, separate null model analyses were conducted for four groups: (i) uncovered soils, (ii) sheep dung-covered soils, (iii) cattle dung-covered soils, and (iv) horse dung-covered soils. Within each group, pairwise comparisons were made across all seven depth layers (0–5, 5–10, 10–20, 20–30, 30–50, 50–70, and 70–100 cm). The β-mean nearest taxon distance (β-MNTD) between soil samples and its null distribution was calculated in R using the comdistnt function (picante package). The deviation between observed and null expectations was quantified by calculating the β-nearest taxon index (β-NTI). We also calculated the Raup–Crick metric based on Bray–Curtis dissimilarity (RC_Bray_) to compare the observed community dissimilarity against a null model. RC_Bray_ was interpreted jointly with βNTI to evaluate the community assembly process [45]. Based on established thresholds, pairwise community comparisons were categorized according to their β-NTI and RC_Bray_ values: heterogeneous selection was inferred when β-NTI > 2, whereas homogeneous selection was indicated by β-NTI < −2. For comparisons where |β-NTI| < 2, dispersal limitation was assigned if RC_Bray_ > 0.95, and homogenizing dispersal was assigned if RC_Bray_ < −0.95. Finally, pairwise comparisons exhibiting |β-NTI| < 2 and |RC_Bray_| < 0.95 were attributed predominantly to undominated, which is also described as a ‘drift’ assembly process.

## 3. Results

### 3.1. Shifts in Soil Microbial Community Diversity and Structure Induced by Dung Deposition

Through the current sequencing efforts, the rarefied sequences were clustered into 4532 bacterial, 2889 fungal and 150 archaeal OTUs at 97% identity thresholds. Alpha diversity showed distinct responses to dung deposition depending on dung source, with the most pronounced shifts observed in cattle dung-covered soils (Table 1). To be specific, the α-diversity index of bacterial communities, such as the richness index (observed number of OTUs, Chao 1 and ACE estimator) and diversity index (Shannon, Simpson and phylogenetic diversity indexes), exhibited no significant variation among the dung-covered and uncovered soils. Fungal communities exhibited significant increases in the Chao 1 estimator and ACE index in sheep dung-covered soils but significant decreases in the Shannon and Simpson indexes in cattle and horse dung-covered soils. Archaeal communities showed the most significant differences in cattle dung-covered soils, where richness estimators (observed number of OTUs and Chao 1) and diversity indexes (ACE and PD) were significantly decreased compared to uncovered soils.

**Figure 1:**
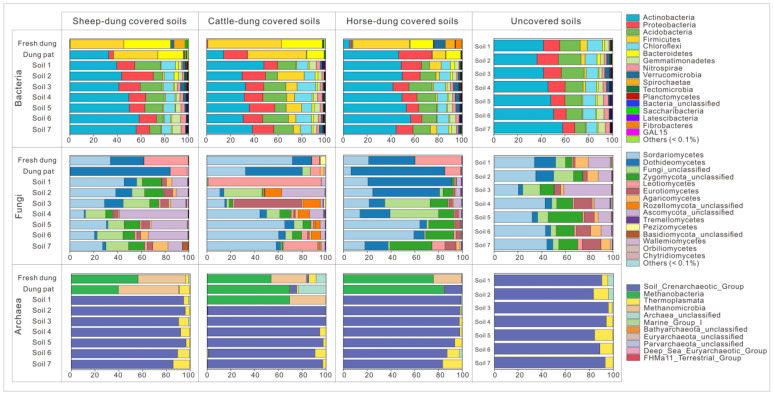
Overview of the microbial taxonomic information of fresh dung, air-dried dung pats and dung-covered soils. Bacterial communities were visualized at the phylum level, while fungal and archaeal communities were visualized at the class level. Soil 1: soils collected from 0–5 cm; Soil 2: 5–10 cm; Soil 3: 10–15 cm; Soil 4: 15–20 cm; Soil 5: 20–30 cm; Soil 6: 30–50 cm; Soil 7: 50–70 cm.

The composition of the microbial community in dung-covered soils was distinct from fresh dung or dung pats at the phylum or class level (Figure 1). In the fresh dung of livestock and dung pats, prokaryotic communities were dominantly composed of the bacterial phyla Firmicutes and Bacteroidetes, as well as methanogenic groups, including Methanobacteria and Methanomicrobia, while fungal communities were predominated by the classes Sordariomycetes, Leotiomycetes and Dothideomycetes. The soil bacterial community was dominated by the phylum Actinobacteria (45% on average), followed by Proteobacteria (15%), Acidobacteria (15%) and Chloroflexi (10%), while the soil fungal and archaeal communities were dominated by the class Sordariomycetes (33%) and the Soil Crenarchaeotic Group (90%), respectively. Venn diagram analysis revealed that 27%, 43%, and 49% of the soil microbial genera in sheep, cattle, and horse dung-covered soils, respectively, were also present in fresh dung or dung pats (Supplementary Figure S1). Among these dung–soil co-occurring genera, only 46 bacterial, 25 fungal, and three archaeal genera were absent from control (uncovered) soils (Supplementary Figure S2).

PCoA analysis was utilized to depict the shifts in soil microbial community structure among different dung-covered soils (Supplementary Figure S3). The dissimilarity tests based on Bray–Curtis distances showed that cattle dung deposition-induced community coalescence tended to be associated with significant structural changes in all three microbial domains (Table 2). In contrast, horse dung deposition only altered the structure of soil fungal communities (PERMANOVA, *p* = 0.005; MRPP, *p* = 0.001), and sheep dung deposition did not significantly affect any of the three microbial domains (PERMANOVA and MRPP, *p* > 0.05). When we analyzed the data by soil depth, we found that dung deposition-induced community coalescence resulted in larger structural shifts in deeper soils than in shallow soils (Supplementary Figure S4 and Table S1).

### 3.2. Correlation Between Edaphic Properties and Soil Microbial Community Structure

**Figure 2:**
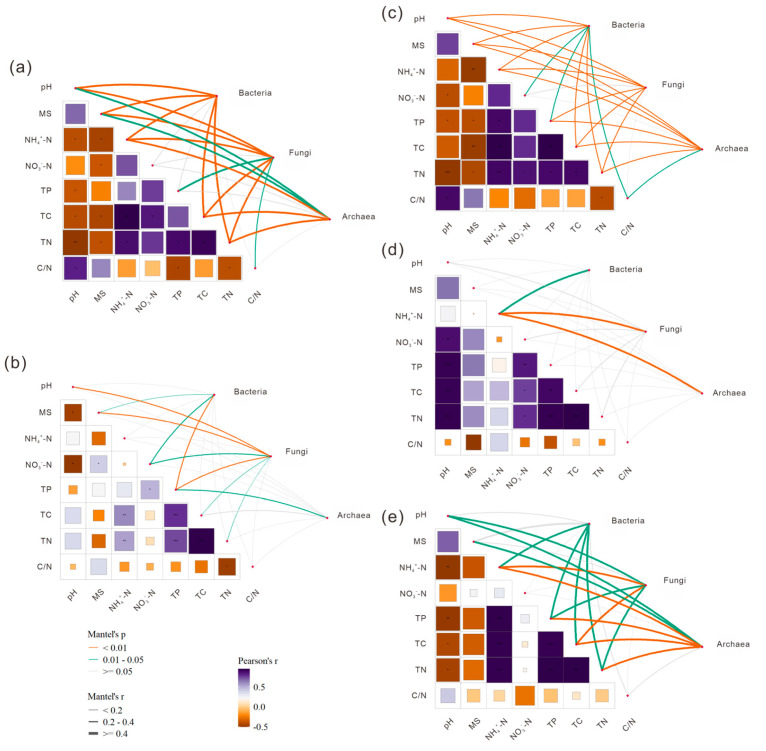
The relationships between soil microbial community and edaphic properties for uncovered (**a**) and dung-covered soils (**b**). Panels (**c**–**e**) represent the correlations between the soil microbial community and edaphic properties for sheep, cattle and horse dung-covered soils, respectively. MS: soil moisture; TP, total phosphorus content; TC, total carbon content; TN: total nitrogen content; C/N: the ratio of total carbon and nitrogen. * *p* < 0.05, ** *p* < 0.01, *** *p* < 0.001.

Overall, several edaphic properties contributed to explaining the variation in soil microbial community structure in both dung-covered and uncovered soils, but community coalescence induced by dung deposition generally weakened these associations (Figure 2a,b). However, when we separated dung-covered soils by livestock species, clear differences emerged between them (Figure 2c–e). For sheep dung-covered soils, dung deposition exerted limited effects on the relationship between soil microbial communities and edaphic properties. In contrast, cattle and horse dung deposition weakened the relationships between the variation in soil microbiota and edaphic properties to different degrees. Notably, in cattle dung-covered soils, only soil NH_4_^+^-N remained closely correlated with microbial community variation.

### 3.3. Shifts in Soil Microbial Co-Occurrence Patterns Driven by Dung Deposition

**Figure 3:**
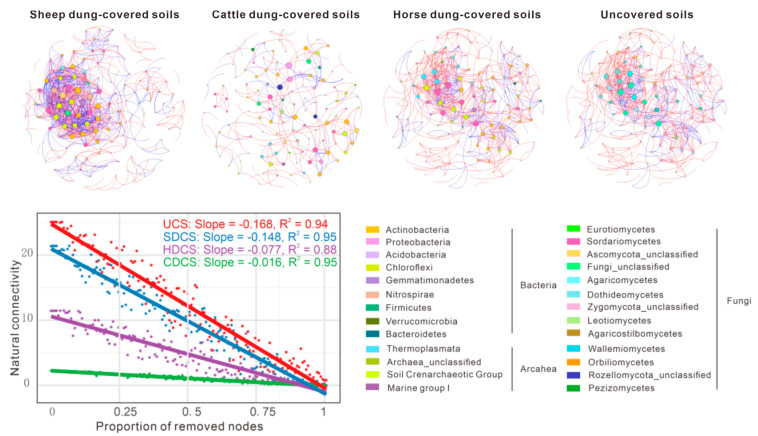
The co-occurrence networks of soil microbial communities and the relationship between natural connectivity and the proportion of removed nodes in the networks. Nodes in the upper panels indicate taxonomic affiliations at the OTU level. The size of each node is proportional to the number of degrees, and the color of each node represents different taxonomic information. Links between nodes indicate significant correlations between those OTUs, and links with red and blue lines in the network represent positive and negative correlations, respectively. SDCSs, sheep dung-covered soils; CDCSs, cattle dung-covered soils; HDCSs, horse dung-covered soils; UCSs, uncovered soil.

To elucidate microbial co-occurrence patterns, we constructed co-occurrence networks for four categories, including sheep dung-covered soils, cattle dung-covered soils, horse dung-covered soils and uncovered soils, resulting in 157, 151, 152 and 167 nodes, respectively (Figure 3). In all four networks, the majority of the connections were affiliated with the bacterial phylum Actinobacteria (18–23%), the fungal class Sordariomycetes (16–21%) and the archaeal class Soil Crenarchaeotic Group (10–11%), but the networks exhibited distinct topological properties. Compared with uncovered soils, networks of dung-covered soils had fewer nodes, fewer edges, and lower density, indicating reduced topological complexity. They also exhibited lower clustering coefficients but higher average path lengths, suggesting weaker and less efficient co-occurrence patterns. In particular, cattle dung-covered soils presented the lowest average degree but the highest average path length and modularity, indicating a weakened microbial co-occurrence pattern with a highly modular structure (Table 3). Moreover, the network robustness of dung-covered soils was lower than that of uncovered soils based on the correlations between microbial natural connectivity and proportion of removed nodes (Figure 3).

To assess the impact of dung deposition-induced community coalescence on microbial interactions from different soil depths, we split the dataset of microbial communities in dung-covered soils and constructed microbial community networks for shallow soils (0–20 cm) and deep soils (20–100 cm). The shallow-soil network exhibited lower complexity (average degree) and lower robustness than the deep-soil network. Additionally, the shallow-soil network had a predominance of positive co-occurrence (91%) and higher modularity, indicating highly cooperative microbial interactions and strong modularization in shallow soils (Supplementary Figure S5 and Table S2).

### 3.4. Community Assembly of Soil Microbial Communities

**Figure 4:**
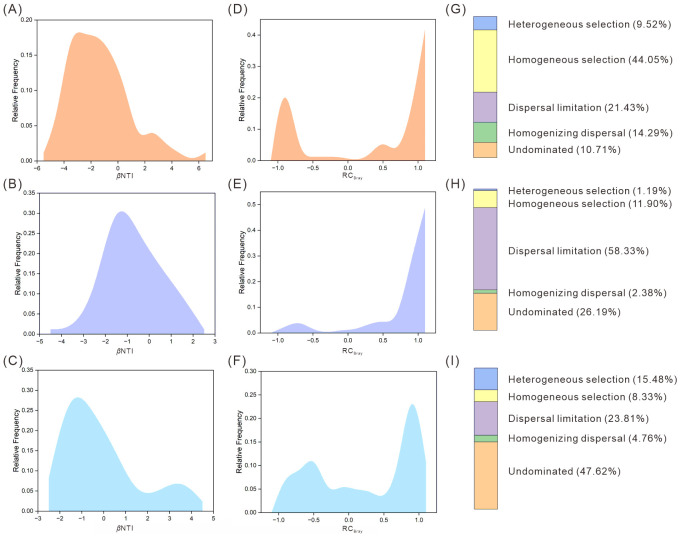
The distribution of β-NTI (panels (**A**–**C**) represent bacteria, fungi, and archaea, respectively) and RC_Bray_ (panels (**D**–**F**) represent bacteria, fungi, and archaea, respectively), and the relative contribution of the ecological processes in governing the community assembly of bacteria (**G**), fungi (**H**), and archaea (**I**) for soil microbial communities.

To determine the dominant process governing soil microbiota assembly, βNTI and RC_Bray_ were calculated for both dung-covered and uncovered soils. As shown in Figure 4, the bacterial phylogenetic turnover differed significantly from the null model in half of the soil pairs (βNTI < −2 in 44.05% of cases; βNTI > 2 in 9.52%). Among the remaining pairs, nearly one-third showed higher taxonomic turnover than expected by chance (RC_Bray_ > 0.95). These results indicate that homogeneous selection dominated bacterial assembly in most soil pairs, whereas dispersal limitation contributed notably to taxonomic turnover in others. Conversely, fungal communities for most soil pairs (87%) did not vary significantly from the null model (|βNTI| < 2). Of these, 67% exhibited higher taxonomic turnover, while 30% showed no significant variations, implying that dispersal limitation and drift tend to be the primary drivers of fungal assembly. For archaea, phylogenetic turnover did not differ significantly from the null model in 76.19% of soil pairs. Among these, nearly 62.5% also showed no significant difference from the null model, indicating that stochastic processes, particularly drift, dominate archaeal phylogenetic turnover.

**Figure 5:**
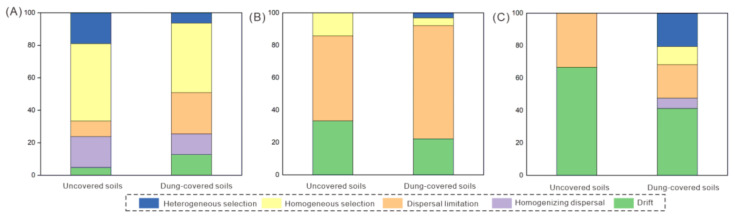
The distribution of the ecological processes that govern community assembly of soil bacteria (**A**), fungi (**B**), and archaea (**C**) between dung-covered soils and uncovered soils.

The effect of dung deposition on assembly processes varied among microbial domains (Figure 5). For bacteria, deterministic processes accounted for 67% of the assembly in uncovered soils, but dung deposition decreased the proportion of deterministic processes while increasing the proportions of dispersal limitation and drift. For fungi, uncovered soils were dominated by stochastic processes (85%), and dung deposition further increased the proportion of stochastic processes at the expense of deterministic processes (specifically homogeneous selection). For archaea, uncovered soils were entirely governed by stochastic processes; however, dung deposition decreased the proportion of dispersal limitation and drift and increased the proportion of deterministic processes (heterogeneous and homogeneous selection).

## 4. Discussion

The consequences of grazing on soil microbial diversity and community structure are well documented [46,47]. However, most studies focused on the overall impacts of grazing practice, whereas the specific ecological impacts of dung deposition-induced community coalescence have received limited attention. The objective of this study was to examine how dung deposition alters soil microbial diversity, community structure, and co-occurrence patterns across different dung sources and soil depths and to elucidate the underlying assembly mechanisms. Consistent with our first hypothesis, dung deposition exerted divergent effects on soil microbial α-diversity depending on dung source and microbial domain (Table 1). The observed changes in microbial diversity reflect the offset between decreasing and increasing impacts of community coalescence induced by dung deposition on soil microbial diversity. Specifically, dung deposition enriches the soil microbial community by introducing dung-derived microorganisms, which may increase microbial diversity [10,19,48]. In addition, short-term dung deposition provides immediate fertility benefits—primarily through increased availability of organic carbon, nitrogen, and phosphorus—thereby promoting the rapid proliferation of copiotrophic microorganisms adapted to nutrient-rich environments. In contrast, a selective pressure through altered edaphic properties and antibiotic agents leads to the loss of some native species [49,50]. Notably, fungal and archaeal communities showed greater sensitivity to dung deposition than bacteria, though the direction and magnitude of these changes varied with dung sources. We attribute this difference to growth characteristics and adaptive mechanisms: dung deposition altered oxygen availability and moisture levels in underlying soil, and the growth of fungal hyphae and the anaerobic preference of methanogens enabled them to respond rapidly to such changes [51,52]. These findings suggest that soil fungal and archaeal community diversity may serve as more sensitive indicators than bacteria for evaluating the impact of dung-induced community coalescence on soil microbial α-diversity. Notably, several microbial taxa were exclusively detected in dung-covered soils but were completely absent from control soils, providing strong evidence for the direct introduction of livestock gut microbiomes via dung deposition. For example, the cellulolytic bacterium *Ruminiclostridium* [53,54], the archaeal lineage Methanobacteriaceae [55] and the coprophilous fungus *Strattonia* [56] are well-documented members of the ruminant gut or dung microbiota. The presence of these taxa in dung-covered soils and their absence in control soils confirm that they are not native to the soil environment but are instead allochthonous microorganisms imported via dung deposition.

Dung-induced community coalescence also altered soil microbial community structure, with the strongest effects observed for cattle dung. The variations in microbial community diversity and structure among dung-covered soils could be attributed to the differences in dung characteristics among livestock species (such as moisture and nutrient composition) as well as the altered edaphic properties caused by dung deposition [22,57]. Moreover, cattle deposit dung in large amounts in a few patches, whereas sheep and horses produce small pellets that are widely distributed. Such variations in the pattern of dung deposition might strengthen the differences in soil microbial community structure among livestock species [58]. A particularly novel finding emerged from the depth analysis: the differences in microbial community structure between dung-covered and dung-free soils were more pronounced in deeper layers (>20 cm) than in shallow soils. This suggests that grazing practices may increase the resistance of soil microbial communities to dung deposition in shallow soil layers. For instance, priority effects (leading to invasion resistance of the soil community) or long-term environmental exposure may confer greater invasion resistance to surface soil communities [18,59], but further experimental tests are still required to confirm this speculation.

The soil microbial community structures showed a strong correlation with edaphic properties in uncovered soils, but the correlation shifted in magnitude and direction under different types of dung deposition—consistent with the general finding that soil microbial community structure is jointly shaped by edaphic properties, land use, and geographical distance [60,61]. For sheep dung-covered soils, the correlations remained largely unchanged—consistent with the absence of significant structural shifts. In contrast, for cattle and horse dung-covered soils, microbial community structure became decoupled from edaphic properties and appeared more closely associated with other factors, such as biotic interactions among soil microorganisms [62,63]. This decoupling implies that dung deposition can override environmental filtering in certain contexts, shifting the primary drivers of community variation.

Microbial co-occurrence patterns have been reported to be sensitive to anthropogenic disturbances and environmental conditions [64,65]. The co-occurrence network analysis confirmed that community coalescence induced by dung deposition consistently reduced the complexity and robustness of the network of soil microorganisms. The results were consistent with previous studies, which suggested microbial interactions might be strongly impaired in copiotrophic environments [66,67], i.e., accelerated nutrient input caused by dung deposition in this case. We further found a higher positive association in shallow soils than in deep soils, indicating that soil microbes may adapt to dung deposition via cooperative interactions. This pattern supports the stress gradient hypothesis, where microorganisms can regulate co-occurrence patterns by decreasing negative co-occurrence associations (e.g., competition) and increasing positive ones (e.g., cooperation) as one of the effective survival strategies under stress [68,69]. Notably, cattle and horse dung-covered soils exhibited increased positive associations and modularity, indicating enhanced positive co-occurrence associations and a highly modular structure that may facilitate adaptation to habitat heterogeneity induced by dung deposition [70,71]. Since the findings could imply the adaptability and survival strategies of soil microbiota when dung-induced community coalescence occurs, we proposed that soil microorganisms enable adaptation to the alterations in environmental conditions caused by dung deposition by regulating their co-occurrence patterns [69,70] as a strategy in the Xilingol grassland steppe.

Turning to assembly mechanisms, we observed contrasting patterns among microbial domains. In general, deterministic processes dominated bacterial assembly (53.7%), contrasting with the strong stochastic influence on fungal (86.9%) and archaeal (76.2%) communities. As shown in numerous previous studies, the relatively weak deterministic (i.e., heterogeneous and homogeneous selection) effect on fungal and archaeal communities may be attributed to their high adaptability to survive in varied environments [72,73]. Importantly, dung deposition altered the balance of assembly processes in domain-specific ways. Dung-induced community coalescence increased the relative contribution of the stochastic process to bacterial and fungal communities but increased the deterministic process of the archaeal community. Previous studies have reported that stochasticity might be increased under low ecological pressure, which dampens the influence of environmental filtering [74,75]. The contrasting pattern of microbial assembly among microbial domains may be attributed to their divergent strategies in response to dung deposition. For instance, the majority of bacterial and fungal communities in dung-covered soils are likely adapted to the altered environment (e.g., enhanced nutrient input) resulting from dung deposition. Research has demonstrated that bacteria primarily utilize organic acids and carbohydrates, while fungi are primarily involved in the metabolism of recalcitrant soil carbon (such as residual cellulose and lignin in livestock dung) [76]. However, environmental changes caused by dung deposition might have a negative effect on the survival of the archaeal community. As reported in previous work, the Soil Crenarchaeotic Group (the predominant archaeal class in soils here) was widely distributed in the soil environment and sensitive to soil pH [77,78]; therefore, dung deposition emerged to trigger environmental filtering of soil archaeal communities. Overall, the results demonstrate that dung-induced community coalescence does not uniformly affect assembly processes but rather reconfigures them according to the ecological traits of each microbial domain.

## 5. Conclusions

In conclusion, this study demonstrates that dung deposition significantly reshapes grassland soil microbial communities through community coalescence, with effects that vary by dung source, soil depth, and microbial domain. The results showed that the effects of dung deposition varied among livestock species, though these differences are inherently confounded with physical and chemical dung properties. Cattle dung was associated with the largest observed shifts in soil microbial diversity and exerted stronger effects on soil microbial community structure in all three microbial domains. Further analysis revealed that the microbial community structure in deep soils was more susceptible to the influence of dung deposition than in shallow soils, suggesting a possible higher resistance of shallow-soil microbial communities, though this interpretation remains speculative. Moreover, community coalescence induced by dung deposition led to a less complex and robust microbial co-occurrence network across all dung-covered soils, suggesting a more sensitive response of microbial network attributes than community structure. Based on the ecological process that governs community assembly, we uncovered a contrasting turnover pattern in response to dung deposition among three microbial domains. We found that within the same metacommunity, dung deposition increased the proportion of the stochastic process for the soil bacterial and fungal communities but increased the deterministic process for the archaeal community. These findings advance our knowledge of how dung deposition shifts microbial community structure and interactions, thereby uncovering the distinct ecological processes that assemble soil microbiota in different microbial domains. Further experimental tests are still required to expand our observations to other grassland types and also to dive deep into their impact on grassland functioning.

## Figures and Tables

**Table 1:** Comparison of alpha diversity indexes of microbial communities in different dung-covered and uncovered soils. The values in the table represent mean ± standard error. Values with different letters in the same column indicate significant differences between dung-covered soils and uncovered soils.

	Samples	Sobs	Chao1	ACE	Shannon	Simpson	PD
Bacteria	Uncovered soils	1617.00 ± 77.08 a	2130.74 ± 89.48 a	2128.55 ± 96.22 a	6.15 ± 0.08 a	0.9942 ± 0.0006 a	120.39 ± 4.69 a
	Sheep dung-covered soils	1629.29 ± 85.27 a	2135.09 ± 116.70 a	2141.86 ± 111.20 a	6.10 ± 0.10 a	0.9935 ± 0.0008 a	119.22 ± 5.21 a
	Cattle dung-covered soils	1716.71 ± 54.95 a	2219.60 ± 83.51 a	2221.53 ± 88.36 a	6.19 ± 0.06 a	0.9914 ± 0.0024 a	129.21 ± 3.89 a
	Horse dung-covered soils	1627.14 ± 74.76 a	2161.09 ± 83.30 a	2164.50 ± 84.59 a	6.10 ± 0.08 a	0.9936 ± 0.0005 a	114.41 ± 3.95 a
Fungi	Uncovered soils	538.00 ± 50.93 a	642.11 ± 64.53 a	639.07 ± 64.64 a	3.98 ± 0.12 a	0.94 ± 0.01 a	121.89 ± 7.93 a
	Sheep dung-covered soils	609.00 ± 46.04 a	773.53 ± 74.55 b	762.85 ± 61.87 b	3.88 ± 0.22 a	0.93 ± 0.02 a	129.13 ± 6.50 a
	Cattle dung-covered soils	452.57 ± 68.05 a	528.64 ± 71.63 a	532.51 ± 70.81 a	2.77 ± 0.49 b	0.74 ± 0.11 a	115.47 ± 16.19 a
	Horse dung-covered soils	508.00 ± 22.27 a	621.65 ± 38.98 a	626.08 ± 38.03 a	3.14 ± 0.09 b	0.85 ± 0.01 b	106.71 ± 3.40 a
Archaea	Uncovered soils	61.14 ± 3.18 a	65.72 ± 4.14 a	66.61 ± 4.25 a	2.68 ± 0.06 a	0.8934 ± 0.0085 a	6.58 ± 1.17 a
	Sheep dung-covered soils	65.00 ± 3.22 a	74.98 ± 6.05 a	74.73 ± 5.20 b	2.66 ± 0.04 a	0.8928 ± 0.0049 a	8.13 ± 1.28 a
	Cattle dung-covered soils	44.29 ± 6.38 b	46.83 ± 6.96 b	47.08 ± 6.53 b	2.40 ± 0.15 a	0.8686 ± 0.0217 a	2.43 ± 0.44 b
	Horse dung-covered soils	55.71 ± 2.52 b	61.49 ± 2.05 a	60.86 ± 1.88 a	2.61 ± 0.07 a	0.8868 ± 0.0102 a	4.94 ± 0.62 a

**Table 2:** Dissimilarity test based on permutational multivariate analysis of variance (PERMANOVA) and multiple response permutation procedure (MRPP) between different dung-covered and uncovered soils. SDCSs, sheep dung-covered soils; CDCSs, cattle dung-covered soils; HDCSs, horse dung-covered soils; UCSs, uncovered soils.

	PERMANOVA		MRPP	
	Pseudo F	P	A	P
Bacteria				
SDCSs, CDCSs, HDCSs and UCSs	2.2569	0.01	0.0692	0.007
SDCSs and UCSs	0.5197	0.704	−0.0236	0.749
CDCSs and UCSs	3.3055	0.013	0.0844	0.025
HDCSs and UCSs	1.3217	0.212	0.0203	0.203
Fungi				
SDCSs, CDCSs, HDCSs and UCSs	3.6485	0.001	0.1345	0.001
SDCSs and UCSs	1.7293	0.113	0.0350	0.075
CDCSs and UCSs	4.1893	0.001	0.1132	0.001
HDCSs and UCSs	3.5708	0.005	0.0964	0.001
Archaea				
SDCSs, CDCSs, HDCSs and UCSs	1.6795	0.052	0.0661	0.024
SDCSs and UCSs	0.4403	0.622	−0.0287	0.691
CDCSs and UCSs	2.3539	0.024	0.1016	0.004
HDCSs and UCSs	0.9799	0.394	0.0048	0.330

**Table 3:** Topological features of microbial networks across different dung-covered and uncovered soils.

Topological Properties	Sheep Dung-Covered Soils	Cattle Dung-Covered Soils	Horse Dung-Covered Soils	Uncovered Soils
Number of original OTUs	874	861	845	863
Total nodes	157	151	152	167
Total links	803	240	555	949
Average path length	4.323	7.104	5.474	3.877
Average clustering coefficient	0.615	0.574	0.566	0.617
Average degree	10.229	3.179	7.303	11.365
Density	0.066	0.021	0.048	0.068
Positive co-occurrence (percentage)	53%	71%	72%	59%
Modularity	0.45	0.848	0.65	0.404

## Data Availability

The sequence data obtained in this study were deposited in the NCBI Short Read Archive (SRA) under the Bioproject accession number PRJNA392724, with Biosample numbers SAMN07305413–SAMN07305415.

## Supplementary Materials

The following supporting information can be downloaded at https://www.mdpi.com/article/10.3390/microorganisms14071493/s1, Table S1: Dissimilarity test based on permutational multivariate analysis of variance (PERMANOVA) and multiple-response permutation procedure (MRPP) between different dung-covered and uncovered soil across different depths. SDCS, sheep dung-covered soils; CDCS, cattle dung-covered soils; HDCS, horse dung-covered soils; UCS, uncovered soils. Table S2: Topological features of microbial networks in dung-covered soils across different depths. Figure S1: Venn diagram showing numbers of shared and unique microbial genera between different habitats in sheep dung-covered soils (a), cattle dung-covered soils (b) and horse dung-covered soils (c) and the microbial composition by three major domains. Figure S2: Heatmap showing the co-occurring genera detected in both dung and dung-covered soil samples but absent from uncovered (control) soils, for (a) bacterial, (b) fungal, and (c) archaeal communities. Abundance values are log_2_-transformed. Figure S3: PCoA ordination based on Bray-Curtis dissimilarity for bacteria (A), fungi (B) and archaea (C). Symbols with red down triangles indicate soils from sheep dung-covered soils, green squares indicate cattle dung-covered soils, blue circles indicate soils from horse dung-covered soils, and grey up triangles indicate uncovered soils. Figure S4: PCoA ordination based on Bray-Curtis dissimilarity for bacteria (A,B), fungi (C,D) and archaea (E,F) across different soil depth. The left panels (A,C,E) represent shallow soils and right panels (B,D,F) represent deep soils. Symbols with red down triangles indicate soils from sheep dung-covered soils, green squares indicate cattle dung-covered soils, blue circles indicate soils from horse dung-covered soils, and grey up triangles indicate uncovered soils. Figure S5: Co-occurrence patterns of dung-covered soil microbiota in the shallow (0–20 cm) and deep (20–100 cm) soils. (a) Nodes indicate taxonomic affiliations at the OTU level. The size of each node is proportional to the number of degrees and the colours of nodes represent different taxonomic information (phylum level for bacteria and class level for fungi/archaea). Links between the nodes indicate a significant correlation between those OTUs (*p* < 0.001), and links with red and blue lines in the network represent positive and negative correlations, respectively. (b) Changes in natural connectivity in response to node removal from the networks. Red circles represent microbes in shallow soils and blue circles represents microbes in deep soils.
