# Supplementary material for: Dung-Induced Soil Microbial Community Coalescence Driven by Different Dung Sources: Impacts on Community Shifts and Assembly Mechanisms in Grassland Soils
Source: Microorganisms. 2026 Jul 8;14(7):1493. doi: 10.3390/microorganisms14071493 (PMC13413853; doi:10.3390/microorganisms14071493)
Supplement: Supplementary file 1 [file microorganisms-14-01493-s001.zip › microorganisms-4337348-supplementary.pdf]

Supplementary Materials

Dung-induced soil microbial community coalescence driven by different dung sources: impacts on community shifts and assembly mechanisms in grassland soils

Jie Yang [1, 2], Qi Zhang [2], Bobo Wang [2], Fabrice Ndayisenga [2], and Zhisheng Yu [2, 3*]

[1]*School of Water Resources and Environment, China University of Geosciences (Beijing), Beijing, China;* [2]*College of Resources and Environment, University of Chinese Academy of Sciences, Beijing, P.R. China;* [3]*RCEES-IMCAS-UCAS Joint-Lab of Microbial Technology for Environmental Science, Beijing, China*

*Correspondence should be addressed to:

Prof. Zhisheng Yu

College of Resources and Environment

University of Chinese Academy of Sciences

19 A Yuquan Road, Shijingshan District

Beijing 100049, P. R. China

E-mail: yuzs@ucas.ac.cn

Tel: +86 10 88256057

Fax: +86 10 88256057

Table S1 Dissimilarity test based on permutational multivariate analysis of variance (PERMANOVA) and multiple-response permutation procedure (MRPP) between different dung-covered and uncovered soil across different depths. SDCS, sheep dung-covered soils; CDCS, cattle dung-covered soils; HDCS, horse dung-covered soils; UCS, uncovered soils.

Shallow soils	PERMANOVA		MRPP	
	Pseudo F	P	A	P
Bacteria				
SDCS, CDCS, HDCS and UCS	1.7378	0.055	0.0842	0.070
SDCS and UCS	0.668	0.6	-0.0453	0.6
CDCS and UCS	2.3116	0.1	0.1174	0.1
HDCS and UCS	0.8912	0.4	-0.0067	0.4
Fungi				
SDCS, CDCS, HDCS and UCS	2.9404	0.001	0.2195	0.001
SDCS and UCS	2.301	0.1	0.1275	0.1
CDCS and UCS	2.6174	0.1	0.1462	0.1
HDCS and UCS	3.6204	0.1	0.2077	0.1
Archaea				
SDCS, CDCS, HDCS and UCS	1.3473	0.071	0.0744	0.096
SDCS and UCS	0.3766	0.7	-0.0816	0.7
CDCS and UCS	1.4248	0.1	0.0815	0.1
HDCS and UCS	0.9405	0.3	-0.0018	0.4
Deep soils	PERMANOVA		MRPP	
	Pseudo F	P	A	P
Bacteria				
SDCS, CDCS, HDCS and UCS	4.2549	0.002	0.2134	0.001
SDCS and UCS	0.8911	0.434	-0.0075	0.4363
CDCS and UCS	8.4568	0.031	0.2907	0.029
HDCS and UCS	2.493	0.057	0.1028	0.0538
Fungi				
SDCS, CDCS, HDCS and UCS	4.5300	0.001	0.2392	0.001
SDCS and UCS	2.9598	0.031	0.1163	0.0295
CDCS and UCS	5.9944	0.031	0.2347	0.0293
HDCS and UCS	2.9962	0.027	0.1241	0.0283
Archaea				
SDCS, CDCS, HDCS and UCS	4.6934	0.002	0.2377	0.001
SDCS and UCS	2.9598	0.033	-0.0183	0.6019
CDCS and UCS	5.9944	0.033	0.3719	0.0273
HDCS and UCS	2.9962	0.036	0.0586	0.057

Table S2 Topological features of microbial networks in dung-covered soils across different depths.

Topological properties	Shallow soils	Deep soils
Number of original OTUs	739	764
Total nodes	168	163
Total links	601	761
Average path length	4.375	3.708
Average clustering coefficient	0.556	0.516
Average degree	7.155	9.337
Density	0.043	0.058
Positive co-occurrence	546 (91%)	535 (70%)
Modularity	0.669	0.469

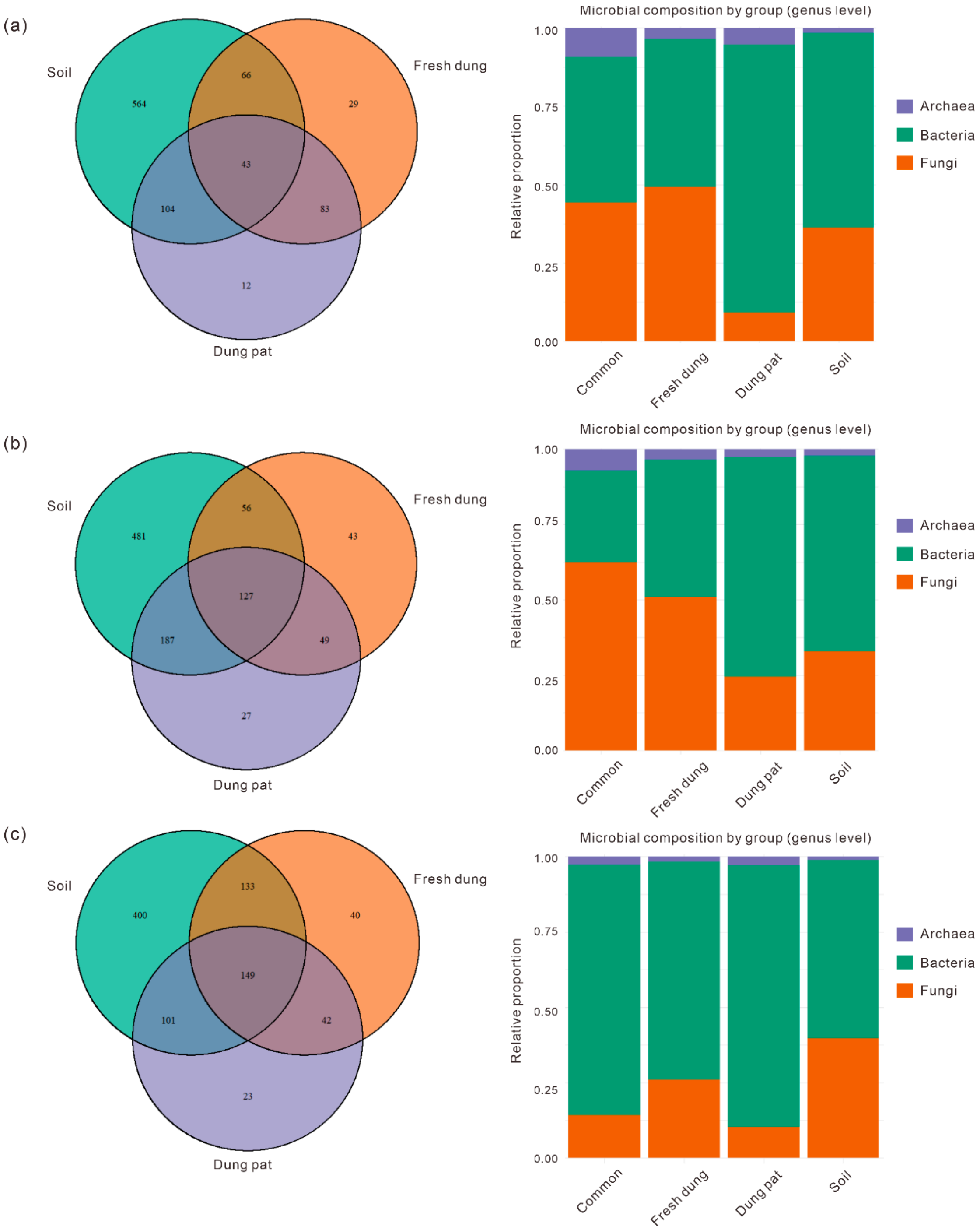

Figure S1 Venn diagram showing numbers of shared and unique microbial genera between different habitats in sheep dung-covered soils (a), cattle dung-covered soils (b) and horse dung-covered soils (c) and the microbial composition by three major domains.

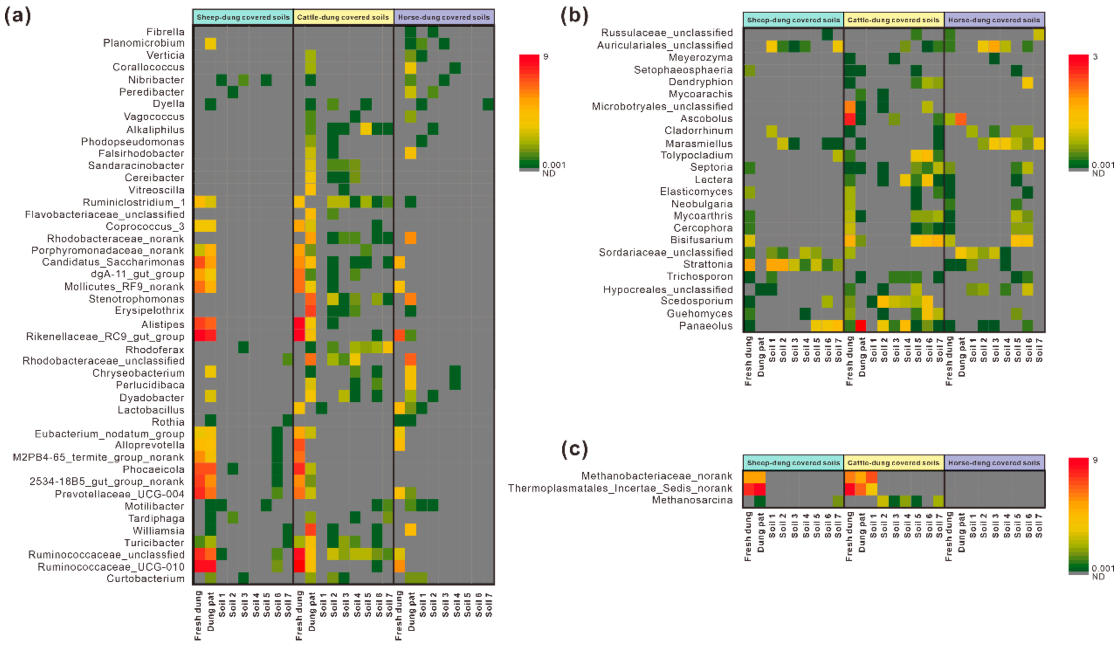

Figure S2 Heatmap showing the co-occurring genera detected in both dung and dung-covered soil samples but absent from uncovered (control) soils, for (a) bacterial, (b) fungal, and (c) archaeal communities. Abundance values are $\log_2$-transformed.

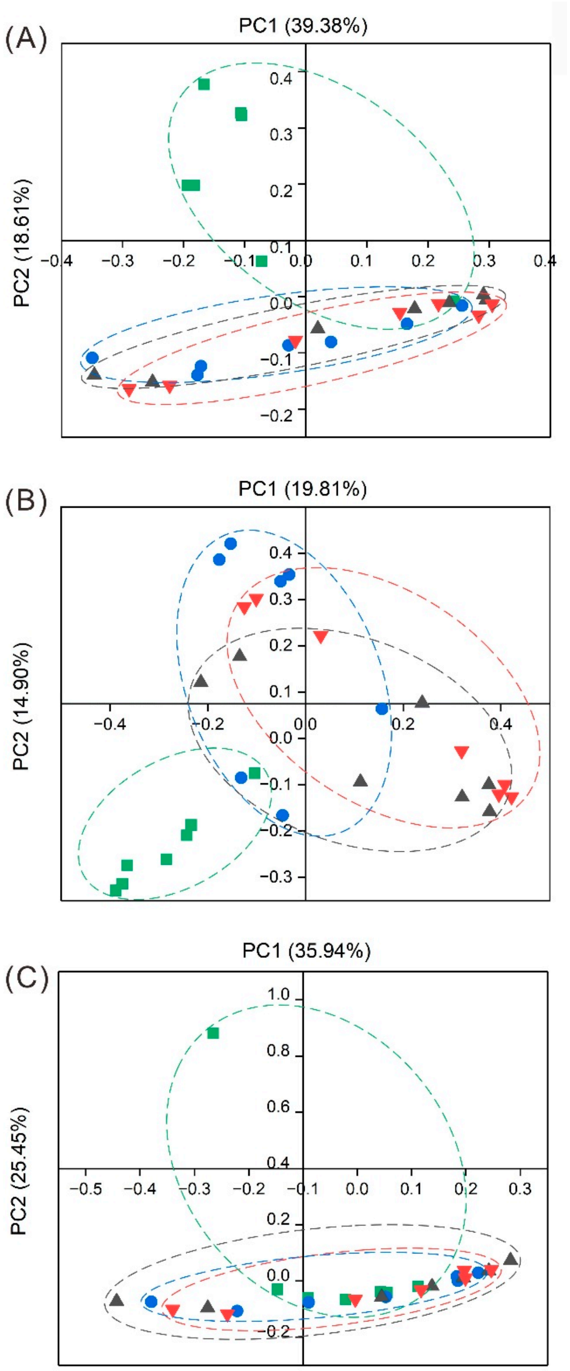

Figure S3 PCoA ordination based on Bray-Curtis dissimilarity for bacteria (A), fungi (B) and archaea (C). Symbols with red down triangles indicate soils from sheep dung-covered soils, green squares indicate cattle dung-covered soils, blue circles indicate soils from horse dung-covered soils, and grey up triangles indicate uncovered soils.

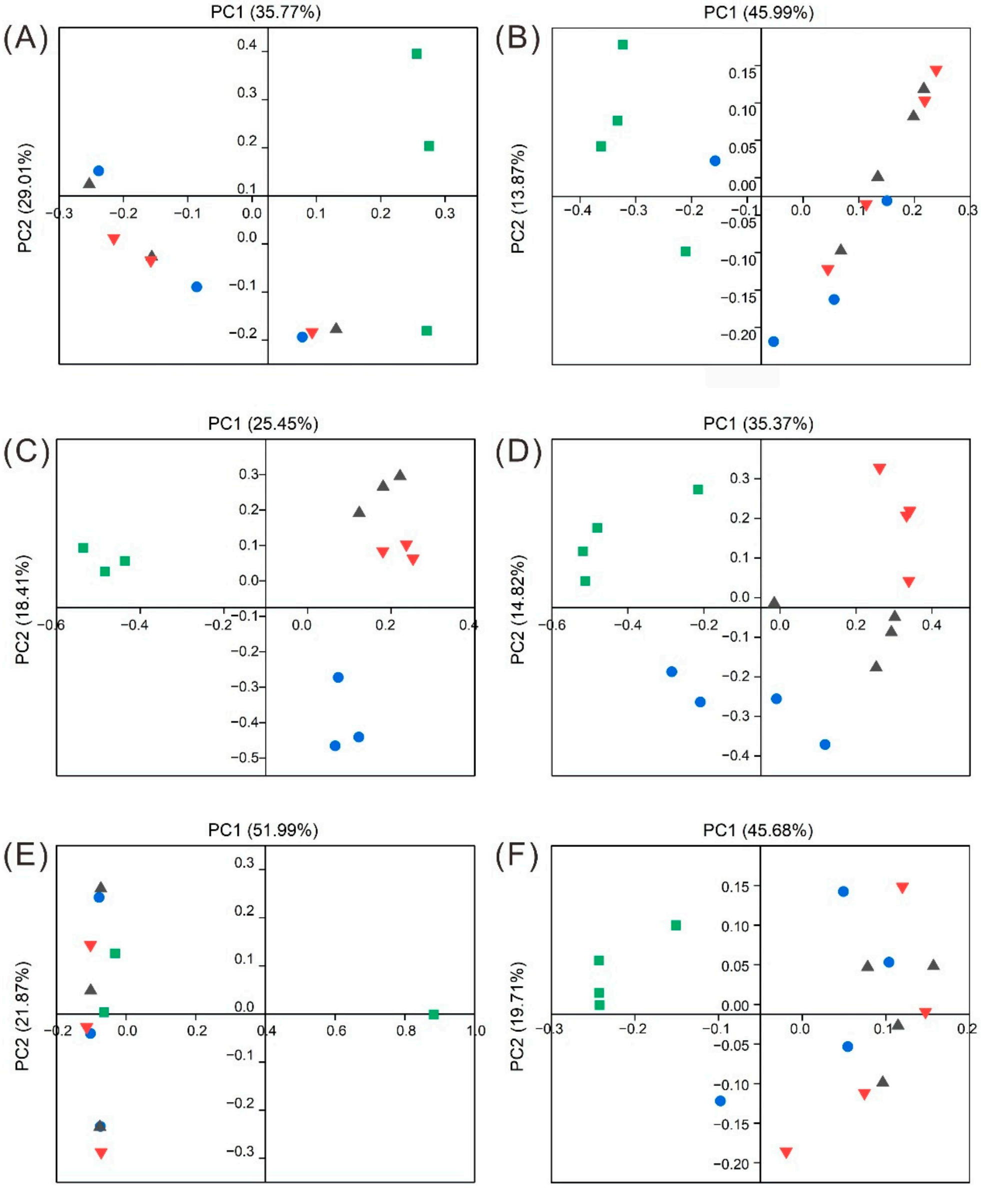

Figure S4 PCoA ordination based on Bray-Curtis dissimilarity for bacteria (A-B), fungi (C-D) and archaea (E-F) across different soil depth. The left panels (A, C, E) represent shallow soils and right panels (B, D, F) represent deep soils. Symbols with red down triangles indicate soils from sheep dung-covered soils, green squares indicate cattle dung-covered soils, blue circles indicate soils from horse dung-covered soils, and grey up triangles indicate uncovered soils.

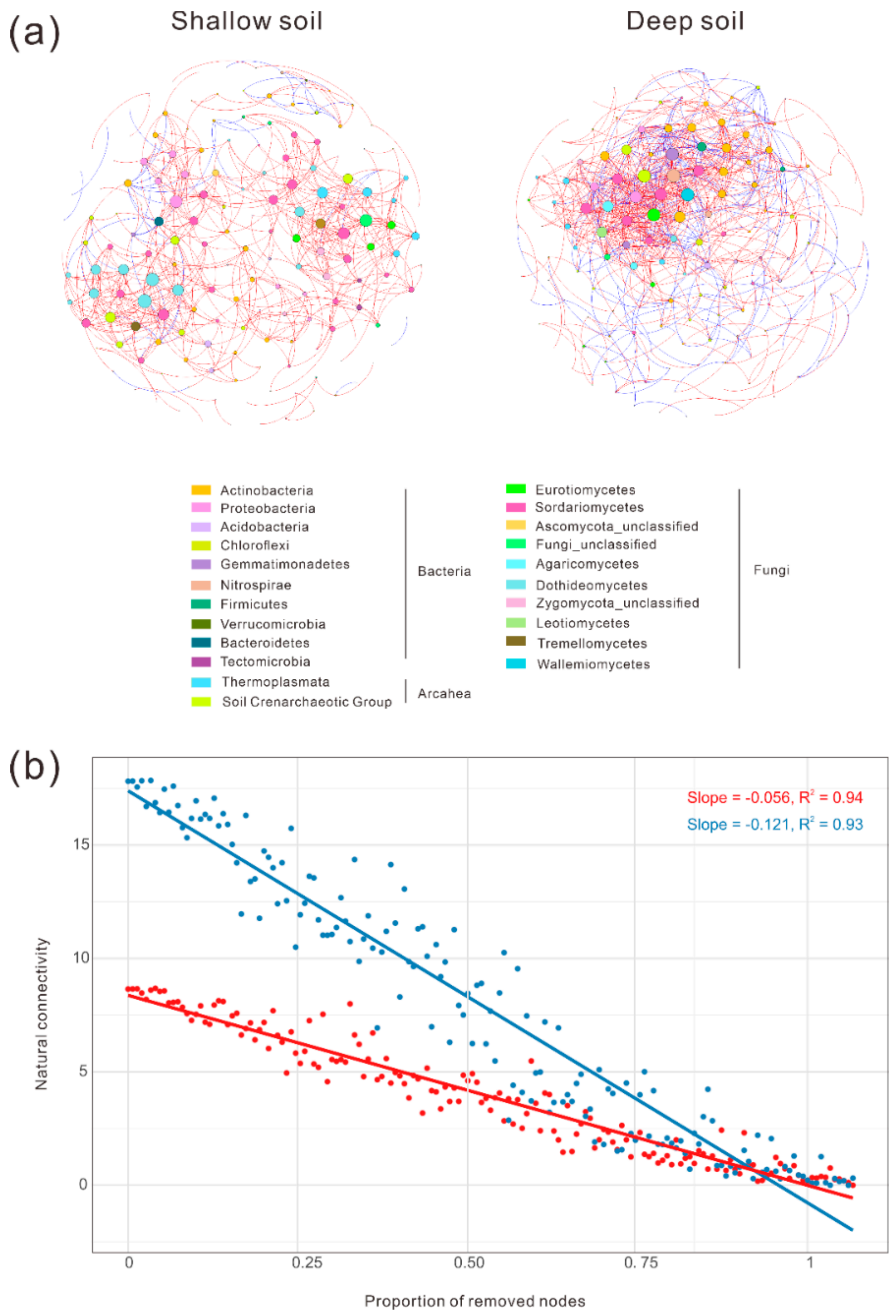

Figure S5 Co-occurrence patterns of dung-covered soil microbiota in the shallow (0—20 cm) and deep (20—100 cm) soils. (a) Nodes indicate taxonomic affiliations at the OTU level. The size of each node is proportional to the number of degrees and the colours of nodes represent different taxonomic information (phylum level for bacteria and class level for fungi/archaea). Links between the nodes indicate a significant correlation between those OTUs ($P<0.001$), and links with red and blue lines in the network represent positive and negative correlations, respectively. (b) Changes in natural connectivity in response to node removal from the networks. Red circles represent microbes in shallow soils and blue circles represents microbes in deep soils.
